# Preparation of Palladium/Silver-Coated Polyimide Nanotubes: Flexible, Electrically Conductive Fibers

**DOI:** 10.3390/ma10111263

**Published:** 2017-11-02

**Authors:** Lushi Kong, Guanchun Rui, Guangyu Wang, Rundong Huang, Ran Li, Jiajie Yu, Shengli Qi, Dezhen Wu

**Affiliations:** State Key Laboratory of Chemical Resource Engineering, Beijing University of Chemical Technology, Beijing 100029, China; konglushi@126.com (L.K.); gchrui@126.com (G.R.); 18660582017@163.com (G.W.); hi4vinton@163.com (R.H.); Liranmaterials@163.com (R.L.); eddieyjj@163.com (J.Y.); wdz@mail.buct.edu.cn (D.W.)

**Keywords:** polyimide, palladium/silver, surface metallization, nanoparticle, nanotube

## Abstract

A simple and practical method for coating palladium/silver nanoparticles on polyimide (PI) nanotubes is developed. The key steps involved in the process are silver ion exchange/reduction and displacement reactions between silver and palladium ions. With the addition of silver, the conductivity of the PI nanotubes is greatly enhanced. Further, the polyimide nanotubes with a dense, homogeneous coating of palladium nanoparticles remain flexible after heat treatment and show the possibility for use as highly efficient catalysts. The approach developed here is applicable for coating various noble metals on a wide range of polymer matrices, and can be used for obtaining polyimide nanotubes with metal loaded on both the inner and outer surface.

## 1. Introduction

Polyimides constitute an important class of polymers that possess excellent thermal stability, wear resistivity, and superior mechanical properties. These outstanding properties have enabled the application of pristine polyimide materials in various fields such as aerospace, defense, and transportation. Several efforts have been undertaken to functionalize or modify polyimide materials and thus extend their applicability into new areas [1,2,3,4,5,6,7,8,9,10]. Surface metallization has attracted great interest for its significant contribution to optics, electrical engineering, magnetism, and chemical catalysis [11,12,13]. Surface metallization is particularly attractive for PI materials as the precursor poly(amic acid) (PAA) contains active carboxyl sites and is highly reactive, thereby enabling facile functionalization [14,15,16,17,18,19,20,21].

Our group has made significant advances in the fabrication of surface-silvered polyimide materials, including films [22], nanocubes [23], and nanofibers [24]. In these studies, the attachment of palladium particles has received less attention because direct metallization with solution containing palladium ions corrode the surface of PAA nanofibers, resulting in a decrease in the number of attachment sites. The high concentration solution containing palladium ions even destroyed the PAA nanofibers (See Supplementary Materials, Figure S3). However, in our recent study on the reaction between silver ions and PAA [25], we have found and confirmed the formation of a cross-linking point among PAA polymer chains during the ion exchange step, which not only makes the attachment of palladium nanoparticles easier, but also strengthens the nanofibers (NF). Inspired by our recent discovery, we report a more efficient method for obtaining Pd/Ag polyimide nanotubes. The nanotube films display high flexibility and large specific surface areas. The addition of silver helps improve the conductivity of the insulating organic matter, and the palladium nanoparticles provide high catalytic activity. These organic-inorganic composite nanotubes may find potential application in catalysis.

## 2. Results and Discussion

**Figure 1:**
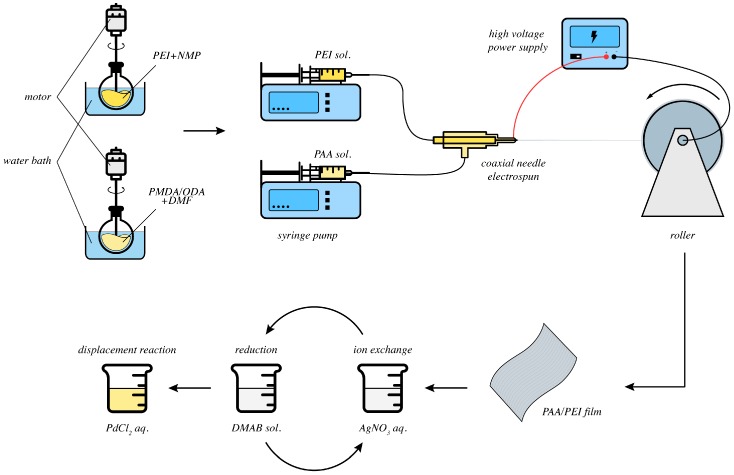
Schematic of the synthesis of our palladium-coated composite material.

Figure 1 depicts the steps involved in the fabrication of the palladium/silver-coated PAA/PEI nanofibers. The first step involved the synthesis of PAA in dimethyl formamide (DMF). This was followed by the fabrication of the coaxial nanofiber film by coaxial electrospinning. Polyetherimide (PEI) was selected as the core because it is compatible with PAA and easy to remove. During the electrospinning process, the intermolecular forces were sufficiently large for maintaining the nanostructure.

### 2.1. Ion Exchange and Reduction

**Scheme 1:**
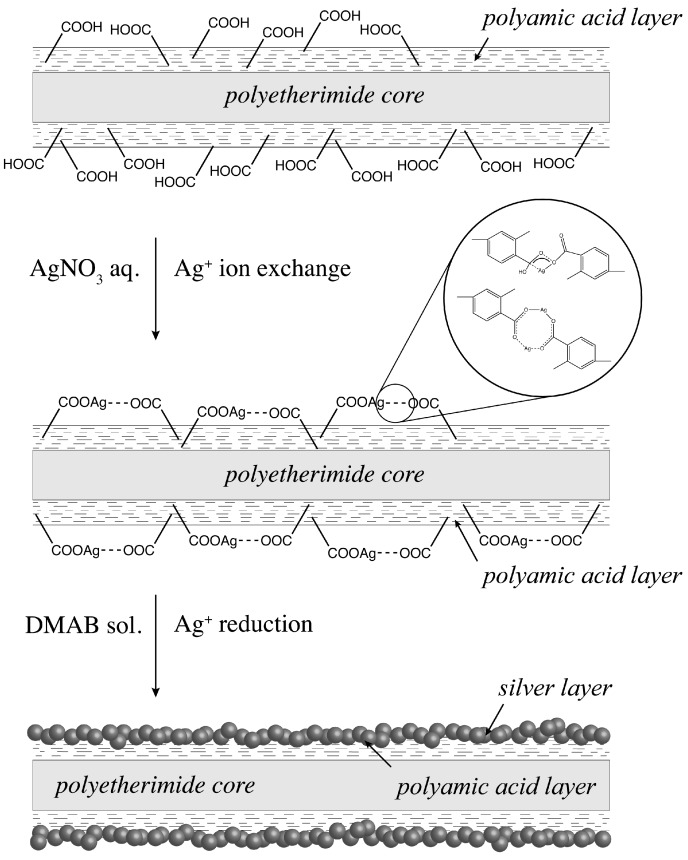
Chemistry involved in the ion exchange of poly(amic acid) with metal ions.

**Figure 2:**
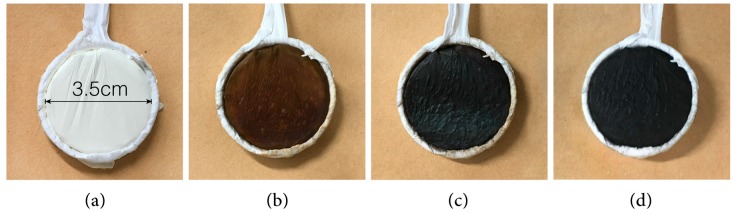
Change in the appearance of the nanofiber films during the metal loading process. (**a**) Untreated poly(amic acid) (PAA)/PEI film; (**b**) PAA/PEI film with AgNO_3_/dimethylamine borane (DMAB) ion exchanged once; (**c**) PAA/PEI film with AgNO_3_/DMAB ion exchanged 4 times; (**d**) metal displacement after 5 h.

The chemistry involved in the silver functionalization is detailed in previous studies [25]. Nevertheless, we note from Scheme 1 that, through the process of ion exchange, PAA and silver ions might promote intermolecular bonding. After the reduction of the silver ions, metallic silver is found to crosslink the polymers chains. Silver nitrate and dimethylamine borane (DMAB) solution are used as the silver ion source and reducing agent, respectively. As shown in Figure 2, the ion exchange was repeated 5 times until the film presented a black color.

**Figure 3:**
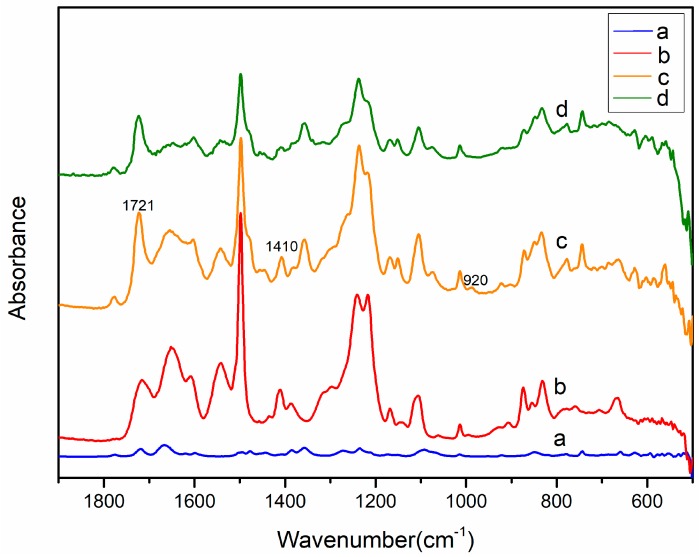
FTIR spectra of (**a**) pure PEI; (**b**) pure PMDA/ODA-based PAA; (**c**) PAA/PEI film; and (**d**) PAA/PEI film after silver ion exchange and DMAB reduction.

Figure 3 shows the Fourier Transform infrared spectroscopy (FTIR) spectra of the pure PEI, pure PMDA/ODA-based PAA, and PAA/PEI film before and after ion exchange. The absorbance spectrum of the PAA/PEI film (Figure 3c) corresponds to a combination of the spectra of the pure PEI (Figure 3a) and pure PAA (Figure 3b). The reaction between PAA and metal ions is verified by comparing spectra c and d. The FTIR spectrum of PAA displays strong bands at 1410 cm^−1^ and 920 cm^−1^, owing to the –OH moiety in –COOH. However, after ion exchange, these bands are weakened, indicating the substitution of the H^+^ cation with silver.

### 2.2. Metal Displacement Reaction

**Figure 4:**
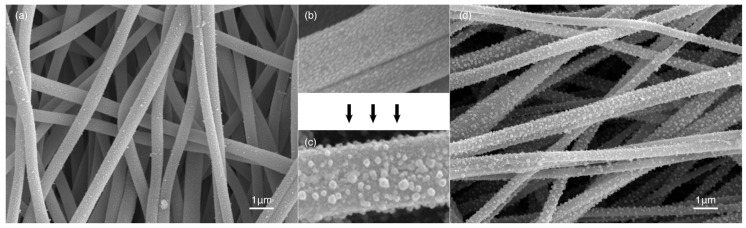
SEM image of nanofibers before and after immersion in 0.5 g/L PdCl_2_ aq. for 1 h. (**a**,**b**) Polyimide nanofibers with silver nanoparticle; (**c**,**d**) the appearance change after PdCl_2_ aq. Treating.

Preliminary experiments were carried out to verify the importance of the silver nanoparticles in the palladium displacement reaction. The silver coated nanofiber film was immersed in palladium chloride solution for a short time (1 h). SEM images depicted in Figure 4 shows the process of palladium initially condenses onto the silver nanoparticle aggregates. Figure 4a,b shows the product we got in Section 2.1, and Figure 4c,d shows the change after pallidium condensinge. We believe the metallic bonds formed between palladium ions and silver are more stable than the ionic bonds between PAA and palladium [13]. We therefore surmise that the silver particles stabilize the attachment of palladium ions on the polymeric nanofiber surface.

Figure 2 records the change in the appearance change of the nanofiber film during metal loading. However, simply increasing the coating time does not produce a contiguous palladium coating. For this, the size and uniformity of the particles must be controlled strictly. Controlled trials were performed with varying Pd^2+^ concentrations and adding potassium bromide, and the results revealed the influence of ion concentration on the particle size. High Pd^2+^ concentrations (PdCl_2_ solution stronger than 4 g/L) led to larger but uneven palladium particles, while low concentrations (PdCl_2_ solution weaker than 0.5 g/L) led to smaller particle sizes and low dispersity. It is reported that potassium bromide controls the crystal morphology of the palladium particles [26]. Palladium nanoparticles barely adhere to the fiber surface in very strong potassium bromide solutions because KBr can disrupt the Pd crystal growth on the (100) crystalline surface. Hence, the maximum concentration of potassium bromide cannot exceed 0.05 M. The results of the abovementioned morphology control experiments are included in Tables S1 and S2 and Figure S5 of the Supplementary Materials.

**Figure 5:**
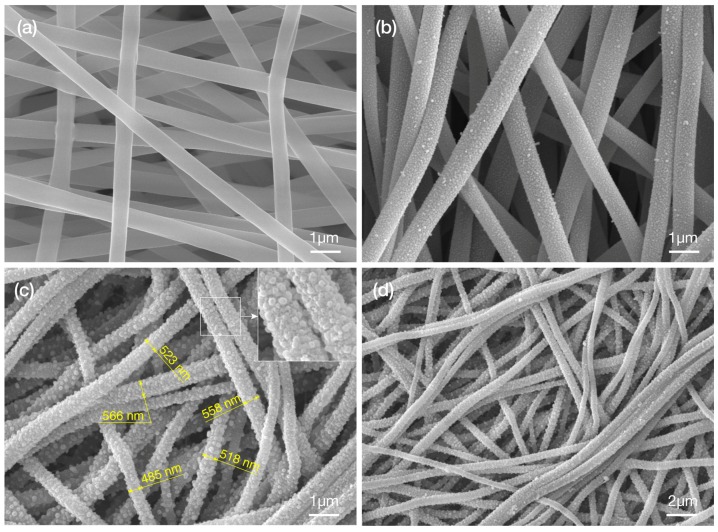
SEM images of (**a**) PAA/PEI nanofibers (**b**) silver-coated nanofibers (NFs), and palladium-coated NFs (**c**,**d**).

Figure 5 shows SEM images of the polymer nanofibers with and without the nanoparticle coating. The average external diameter of the electrospun coaxial nanofibers is about 500 nm, as shown in Figure 5a. Nanofibers decorated with silver nanoparticles are presented in Figure 5b. The inset image in Figure 5c,d show the SEM images of nanofibers after palladium ion displacement, under high and low magnification, respectively.

The SEM images after the individual steps clearly reveal the entire polymer surface metallization process. During the ion exchange and reduction steps, the silver coating is densified, and the size of the Ag particles is sufficiently small for the growth of nano-palladium. This is confirmed by Figure 5c,d, which show that nano-palladium forms a dense layer on the surface of the silver nanoparticles. The low-magnification SEM image in Figure 5d also reveals that the surface metallization reaction is adequate, the entire film is homogeneously coated with a dense, smooth layer of palladium.

**Figure 6:**
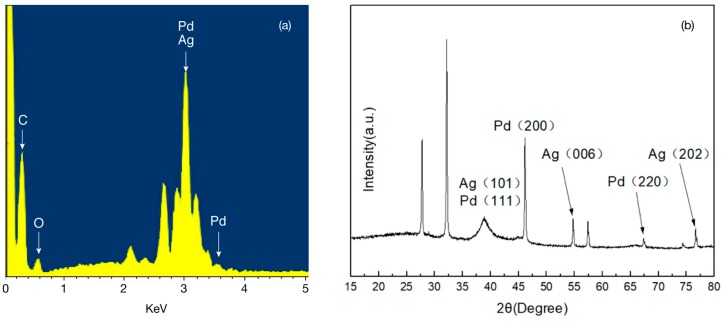
EDS (**a**) and XRD (**b**) analysis of a PAA film after ion exchange in 2 g/L PdCl_2_ aq. for 5 h.

The Energy Dispersive Spectroscopy (EDS) result in Figure 6a shows that in addition to carbon, oxygen, which are typically found in polymers, there is also a high content of silver and palladium. An X-ray diffraction (XRD) pattern typical of palladium crystals is shown in Figure 6b, where diffraction peaks with 2θ values centered at around 46.7° and 68.1° are seen. These peaks correspond to the (200) and (220) crystallographic planes of the fcc structure of Pd, respectively. The XRD pattern also shows diffraction peaks of silver crystals at 55.1° (006) and 76.8° (202). Of equal note is the broad peak near 40.0°, arising from the overlap between the (101) plane of Ag and (111) plane of Pd. The position of several peaks is found to deviate slightly from known peak positions of the pure metals due to the impact of polymer matrix. Additionally, the (100) Pd crystal plane is not observed in the XRD, as it is suppressed by the use of potassium bromide.

### 2.3. Core Displacement and Imidze Reaction

**Figure 7:**
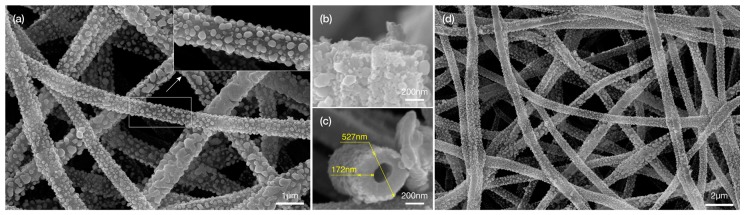
SEM images of palladium-coated PAA nanotubes (**a**,**d**) under different magnifications; (**b**,**c**) views of NTs under high magnification.

To fabricate films with high flexibility and specific strength, further measures for removing the internal PEI core and imidization of the external layer (PAA) were adopted. The film was immersed in NMP (a selective solvent for PEI) at room temperature for 2 days, followed by heat treatment at 300 °C for 2 h for the imidization of PAA into PI. SEM was used to determine the changes in the film morphology after the PEI removal and heat treatment processes. The morphology of a palladium-nanoparticle coated polyimide nanotube film is shown in Figure 7a,d under different magnifications. Figure 7b,c show the detailed structure of a single nanotube. The SEM results indicate that the structure of the superficial nanoparticle coating remains intact after thermal treatment despite the successful removal of the inner layer.

In the coming future, we will carry on to load Pd and Ag nanoparticle on the inner surface of the tube wall using the same way we described in the communication, for which the double-tube-wall loadedd Pd/Ag polyimide which have specific surface area will be obtained.

### 2.4. Electrical Conductivity of Pd/Ag Polyimide Nanotubes

**Figure 8:**
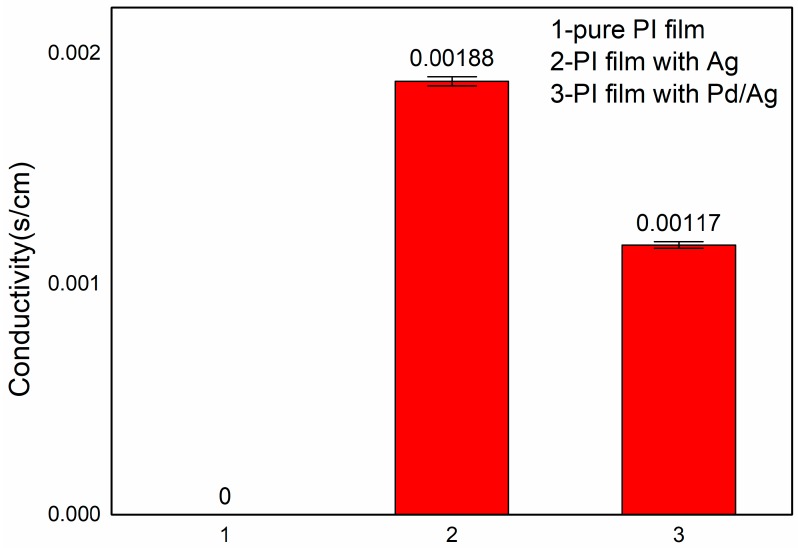
Electrical conductivity of the various films as measured by a four-probe resistivity tester.

Here we demonstrate the electrical conductivity of the Pd/Ag polyimide nanotubes. Figure 8 shows the change in conductivity with Ag and Pd/Ag coating by using a four-probe resistivity tester. The conductivity of the pure PI film is extremely low (about 8 × 10^−16^ s/cm at room temperature) and cannot be detected by a four-probe resistivity tester and therefore indicated as zero in the figure. The conductivity is greatly improved after attaching the silver nanoparticles. Since the conductivity of metallic silver is greater than that of metallic pallidum, the Pd/Ag nanotube film has slightly lower conductivity as compared to the Ag nanotube film.

## 3. Conclusions

In summary, polyimide nanotubes with Pd/Ag nanoparticles were prepared via electrospinning and surface metallization. By coating silver nanoparticles onto the nanotubes, crosslinked sites with improved ability for Pd attachment were achieved. Our technique provides a new avenue for the coating of noble metals such as platinum and gold onto polymeric substrates. Owing to the light weight and flexibility of the polyimide matrix and the electrical conductivity imparted by the nanoparticles, our composite products hold potential for new applications in the field of catalysis. Moreover, the silver coating also led to improved structural stability, which will enable us to prepare polyimide nanotubes with metal loaded in both the inner and outer tube wall in the future.

## Supplementary Materials

The following are available online at www.mdpi.com/1996-1944/10/11/1263/s1, Figure S1: Synthetic scheme of poly(amic acid), Figure S2: Coaxial nanofibers film produced by electrospining, Figure S3: Corrosion of the nanofiber with the method of direct metallization with solution containing palladium ions, Figure S4: Synthesis scheme of palladium loading composite material, Figure S5: Preparation scheme of polyimide, Figure S6: SEM image of different treating methods corresponding to Tables S1 and S2: (a–c) different Pd^2+^ concentration setting separately as 0.5 g/L, 1 g/L and 2 g/L; (d–g): different KBr concentration setting separately as 0.25 M, 0.5 M, 1 M and 2 M, Figure S7: (a) Electrical conductivity of the various films as measured by a four-probe resistivity tester. (b) Cyclic voltammograms of the films in 4 × 10^−5^ M uric acid solution with 0.1 M H_2_SO_4_, obtained at a scan rate of 30 mV/s, Table S1: Treating method of state in Pd^2+^ concentration, Table S2: Treating method of state in different KBr concentration.
