# Supplementary material for: Preparation of Palladium/Silver-Coated Polyimide Nanotubes: Flexible, Electrically Conductive Fibers
Source: Materials (Basel). 2017 Nov 2;10(11):1263. doi: 10.3390/ma10111263 (PMC5706210; doi:10.3390/ma10111263)
Supplement: Supplementary file 1 [file materials-10-01263-s001.pdf]

materials

Supplementary Materials

Methods and Materials

Raw Materials and Solution Preparation

Pyromellitic dianhydride (Tianjin insulating material factory, AR, Tianjin, China), 4,4′-oxydianiline (Shanghai Research Institute of synthetic resin AR, Shanghai, China), N, N-dimethylformamide (Sinopharm chemical reagent Beijing Co., Ltd., AR, Beijing, China), polyetherimide (CR), silver nitrate (Sinopharm chemical reagent Beijing Co., Ltd. AR, Beijing, China), dimethylamine borane (Sinopharm chemical reagent Beijing Co., Ltd., AR, Beijing, China), polyether imide (AR), palladium chloride (Sinopharm chemical reagent Beijing Co., Ltd., AR, Beijing, China), potassium bromide (Sinopharm chemical reagent Beijing Co., Ltd., AR, Beijing, China), ammonia (Sinopharm chemical reagent Beijing Co., Ltd., AR, Beijing, China).

In three neck flasks, ODA (0.0092 mol, 1.8502 g) was added in 20 mL of DMF and stirred to get clear solution. Then PMDA (0.0093 mol, 2.0357 g) was added gradually to the above solution and stirred at ice water bath to get viscous solution. Afterwards, 10 mL of DMF was added to the flask and the resultant solution was stirred for 1h.

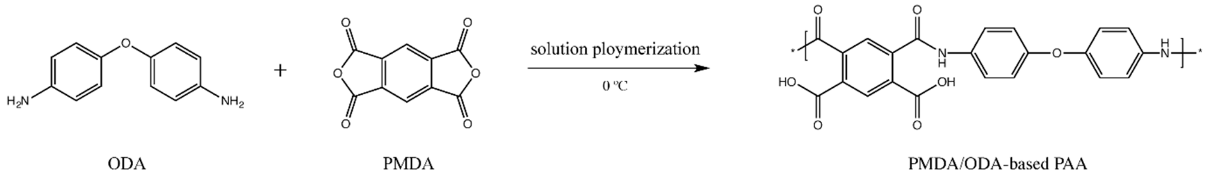

Figure S1. Synthetic scheme of poly(amic acid).

In three neck flasks, 7.4 g polyetherimide was added in 30 mL of NMP and stirred to get clear solution at 60℃ water baths.

Preparation of PAA/PEI Coaxial Nanofiber

Coaxial needle was used to synthesize electrospun coaxial nanofibers, 20 wt % of PEI/NMP solution was linked to inner needle while PAA/DMF to outer.

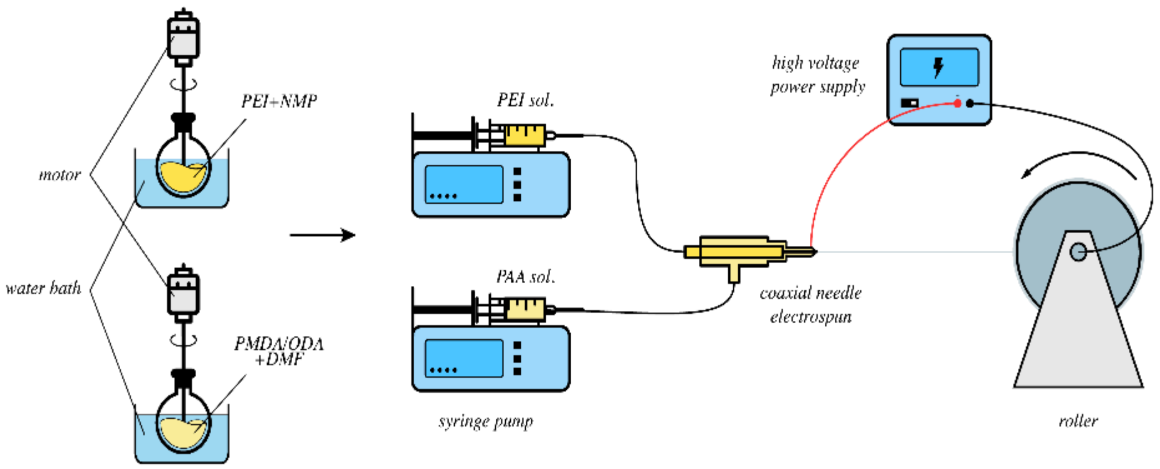

Figure S2. Coaxial nanofibers film produced by electrospining.

Electrospun run for 10 h under 17.2~17.4 KV and 1094 r/min. A white film was obtained.

Preparation of PI Nanotube Coated by Palladium Nanoparticles

The homogeneously palladium nanoparticles coated nanofibers could not easily got by directly metallization. As we mentioned in the commucatuion, "direct metallization with solution containing palladium ions corrode the surface of PAA nanofibers, resulting in a decrease in the number of attachment sites. The high concentration solution containing palladium ions even destroyed the PAA nanofibers."

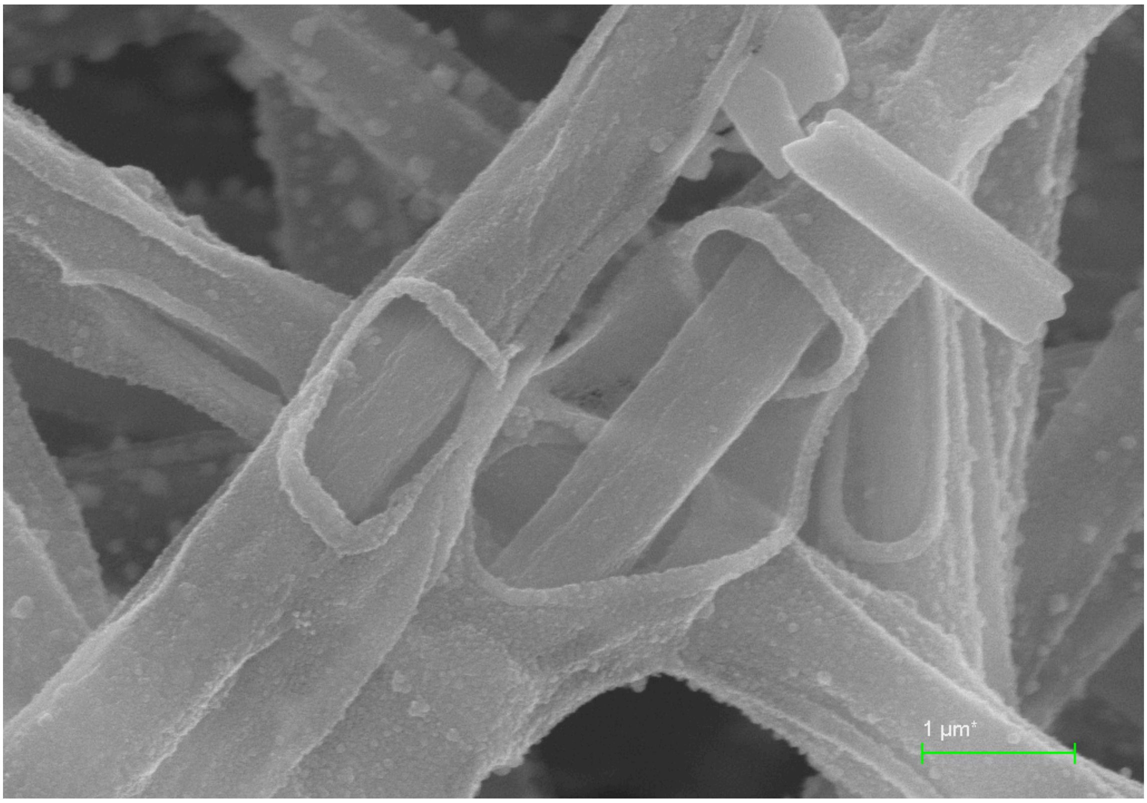

Figure S3. Corrosion of the nanofiber with the method of direct metallization with solution containing palladium ions

PAA/PEI film was immersed into 0.1 M $AgNO_3$ solution for 5 min, cleaned by ultrasonic cleaning for 2 min. Afterwards, the film was immersed into 0.01 M DMAB solution for 5 min and cleaned for 2 min. The above steps were repeated until the film presented black color. Then the black film was immersed into 0.1 M $PdCl_2$ solution at 30 °C for 5 h.

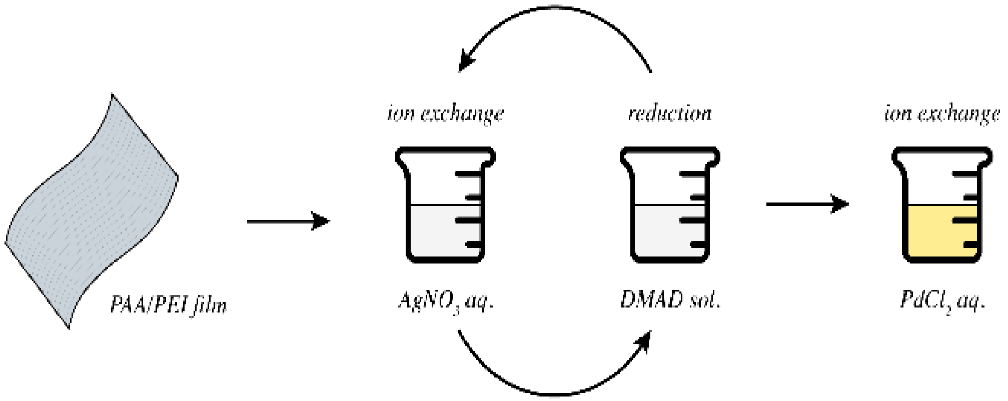

Figure S4. Synthesis scheme of palladium loading composite material.

Firstly, 20 mL NMP was used to remove the PEI inner layer. The PEI inner layer was dissolved after 2 days immersing.

Then, PAA was convert into PI by heat treatment. The temperature was 300℃ and heating rate was 4℃/min, retain the temperature for 2hours. Then slow-air cooling overnight.

Figure S4 shows the reaction in the cyclization reaction.

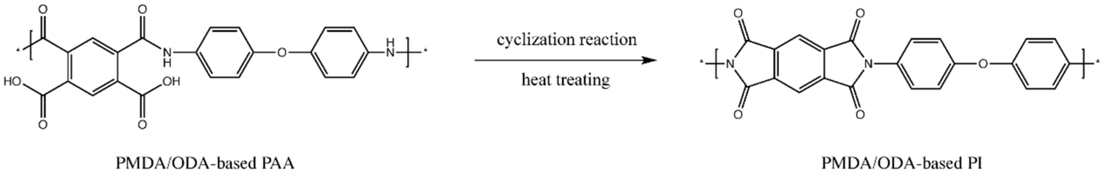

Figure S5. Preparation scheme of polyimide.

Morphology Control

Table S1. Treating method of state in Pd^{2+} concentration.

Sample	T/℃	n^1	C/g/L	t/h
(a)	30℃	5	0.5g/L$PdCl_2$	5
(b)	30℃	5	1g/L$PdCl_2$	5
(c)	30℃	5	2g/L$PdCl_2$	5

1: treating times

Table S2. Treating method of state in different KBr concentration.

Sample	T/℃	n^1	$PdCl_2$/g/L	KBr/M(in 5 mL)	t/h
(d)	30℃	5	2	0.25	5
(e)	30℃	5	2	0.5	5
(f)	30℃	5	2	1	5
(g)	30℃	5	2	2	5

1: treating times

The result correspond to the table is showed in Figure S6.

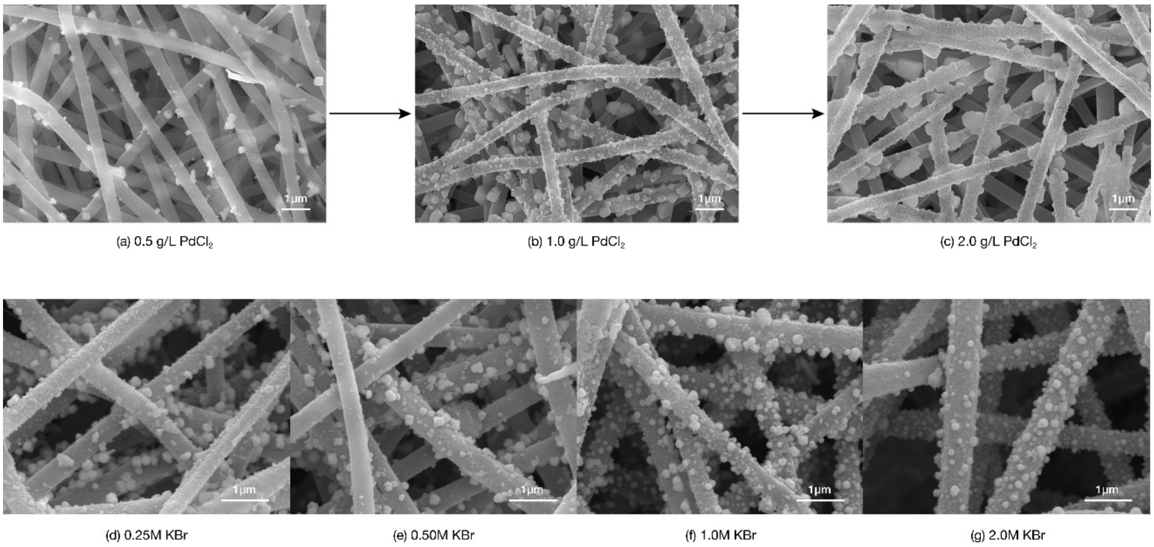

Figure S6. SEM image of different treating methods corresponding to Tables S1 and S2: (**a**–**c**) different Pd^{2+} concentration setting separately as 0.5 g/L, 1 g/L and 2 g/L; (**d**–**g**): different KBr concentration setting separately as 0.25 M, 0.5 M, 1 M and 2 M.

Characterizations

ATR-FTIR spectra were collected using the thermal fisher Nexus 670 IR spectrometer Surface morphology and elemental analysis of the nanofibers was performed on a Zeiss supra 55 field emission scanning electron microscope (FE-SEM) equipped with an energy-dispersive spectrometer produced by Oxford Inc. X-ray diffraction (XRD) experiments were conducted on a Rigaku RINT2200V/PC X-ray diffractometer using CuKα radiation (λ=1.54178) at an accelerating voltage of 40 kV and current of 40 mA.

Catalytic Activity Measurement

Electrochemical measurements were carried out in a three electrode cell at room temperature, chronoamperometric (CA) curves of 3×10^{-5} M uric acid + 0.1 M H_2SO_4 , vs. Hg/Hg_2Cl_2 reference.

The results of cyclic voltammetry (CV) experiments carried out on a pure polyimide film in uric acid (Figure 8(b)) show a maximum current density of −5.7 mA·cm^{-2}. Qualitatively similar behavior is observed with the silver-coated polyimide film but with an increased maximum current density of −9.8 mA·cm^{-2}. Further, within the addition of palladium, a new peak at 0.52 V in the negative sweep appears alongside the silver film current density maximum at 0.65 V, indicating the presence of new redox pathways. Consequently, we note that the silver and palladium nanoparticles play separate but important roles in improving the conductivity and electrocatalytic performance of the Pd/Ag polyimide nanotube films. These results identify our films as excellent organic-inorganic composite material with electrical conductivity and electrocatalytic properties.

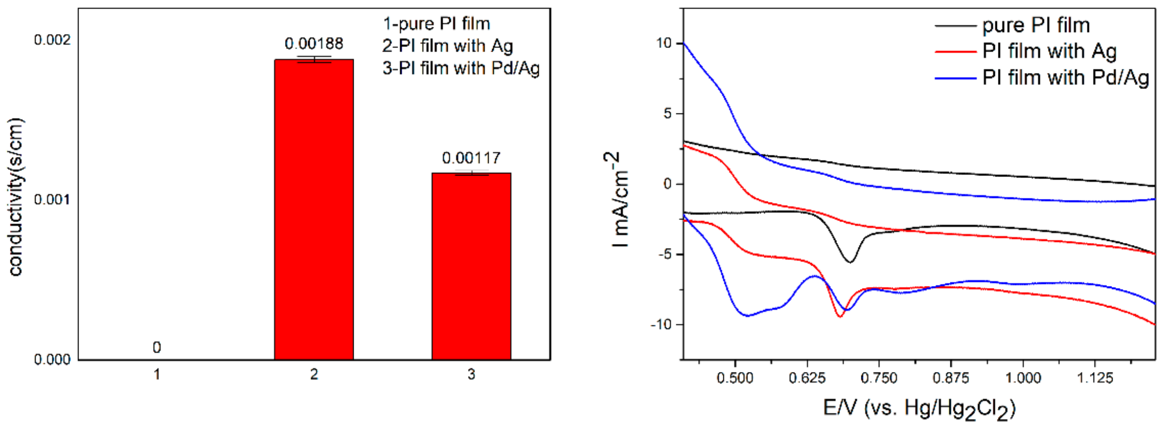

(a) (b)

Figure S7. (**a**) Electrical conductivity of the various films as measured by a four-probe resistivity tester. (**b**) Cyclic voltammograms of the films in 4×10^{-5} M uric acid solution with 0.1 M H_2SO_4, obtained at a scan rate of 30 mV/s.

© 2017 by the authors. Submitted for possible open access publication under the terms and conditions of the Creative Commons Attribution (CC-BY) license (http://creativecommons.org/licenses/by/4.0/).
